# Supplementary material for: Splice-Junction-Based Mapping of Alternative Isoforms in the Human Proteome
Source: Cell Rep. Author manuscript; Available in PMC 2020 Jan 15. (PMC6961840; doi:10.1016/j.celrep.2019.11.026)
Supplement: 3 [file NIHMS1546469-supplement-3.zip › DF2/PXD006675/RightVentricle-393-Q15257-VSEAIEK.pdf]

A

Predicted sequence disorder and sequence features of Q15257

Peptide: VSEAIEK Junction: sp|Q15257|PTPA_HUMAN|ENSG00000119383|SE2|37877|chr9|129123138|129128068|+0|r23|T1 TrNovel: FALSE

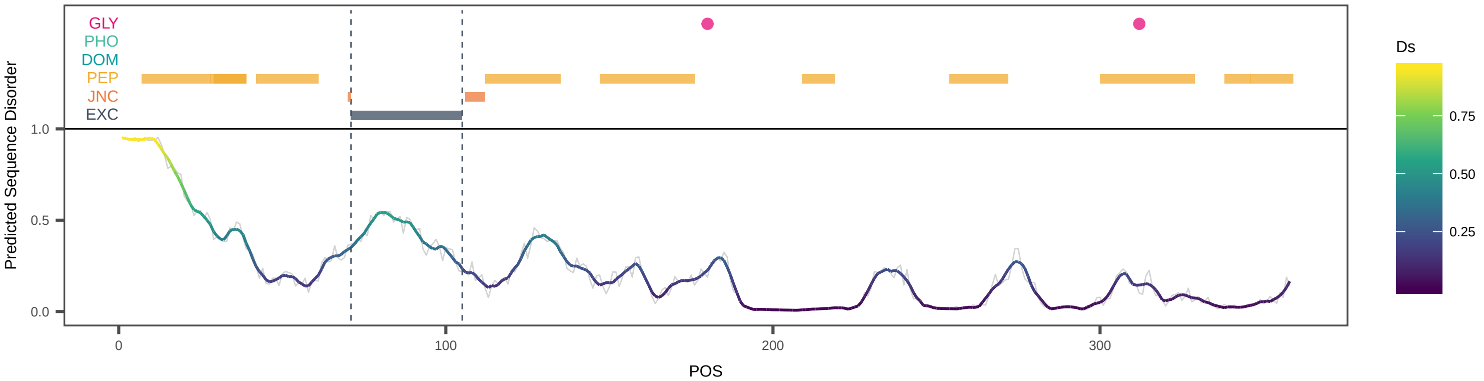

B

Distribution of sequence disorder in excised vs. mapped and non−excised regions of protein

M−W P−value vs. mapped: 9.97e−10 vs. non−excised: 2.75e−13

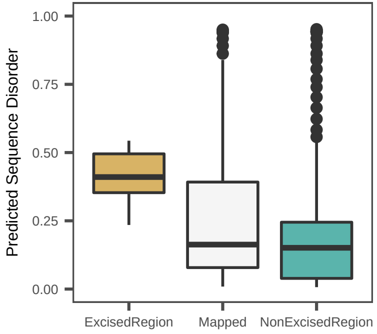

C
